# Preservation of pancreatic endocrine and peri-islet exocrine capillary networks in type 2 diabetes

**DOI:** 10.1172/jci.insight.202118

**Published:** 2026-04-23

**Authors:** Alex M. Tollefson, Frank R. Marsico, Manami Hara

**Affiliations:** Department of Medicine, The University of Chicago, Chicago, Illinois, USA.

**Keywords:** Endocrinology, Vascular biology, Beta cells

## Abstract

Chronic hyperglycemia induces microvascular complications in patients with type 2 diabetes (T2D), particularly diabetic retinopathy, nephropathy, and neuropathy. We revisited the pancreatic vasculature to reexamine such damage in 3D. Using thick pancreatic tissue slices, we analyzed volumetric intraislet capillary density (vICD) and peri-islet volumetric exocrine capillary density (vECD) as well as interface capillary counts along the islet periphery to quantify vascular integration between the islets and surrounding acinar cells. Contrary to the previous reports, vICD was not homogeneous but highly heterogeneous across the five species studied (human, monkey, pig, ferret, and mouse), especially in smaller islets. vICD became less variable with increasing islet size, converging at approximately 20%. With this foundation of islet vascularization, pancreatic tissues from non-diabetic and T2D subjects consisting of 8 age- and sex-matched pairs (age range of 35–65 years with various duration: 0–15 years) were examined. Strikingly, no significant differences in microvascular density were found; mean vICD and mean vECD were nearly equivalent between the groups. Capillary integration with respect to islet size was comparable. These findings suggest that integrated pancreatic blood flow with robust crosstalk between the endocrine and exocrine pancreas may facilitate microvascular preservation in T2D via local distribution of insulin.

## Abstract 2

Using 3D imaging, islet capillary networks were found to be preserved in patients with type 2 diabetes compared with age- and sex-matched non-diabetic controls.

## Introduction

Pancreatic islets have been described as receiving a disproportionately larger blood supply than surrounding exocrine tissues (1), which is considered to be facilitated by markedly denser vasculature than the exocrine regions (2–4). When the islet was believed to be an enclosed micro-organ scattered throughout the pancreas, such an observation was interpreted as indicating a special function of the islet, that its dense vascularization facilitated “closed” and thus “intimate” paracrine effects among various hormone-secreting islet cells. Under the integrated pancreatic blood flow between the endocrine (i.e., islets) and exocrine pancreas that we proposed (5–8), the difference in the density of vascularization can be simply attributed to cell size. β Cells and α cells are about 15 μm in diameter (9), whereas acinar cells are 10–24 μm, peaking at 18 μm (10). It has been noted that when a so-called dogma is established in the field, it has a tremendous impact on all downstream functional sequences.

In type 2 diabetes (T2D), glucose and lipid metabolites adversely promote mechanisms of injury of the vasculature (e.g., advanced glycation end products, reactive oxygen species, angiotensin II, nuclear factor-κB, inflammatory cytokines, leukocyte adhesion, and protein kinase C) and concurrently inhibit protective factors (e.g., insulin, antiinflammatory factors, antioxidant enzymes, platelet-derived growth factor, vascular endothelial growth factor, and activated protein C) (11). An imbalance between molecular mechanisms of damage and endogenous protective factors leads to microvascular complications such as diabetic retinopathy, nephropathy, and neuropathy. There are a large number of mouse models available to investigate diabetes-associated vascular diseases (12). Mouse and rat studies on islet microvascular pathology and inflammation in obesity, T2D, and overnutrition are summarized by Aplin et al. (13). Briefly, all 3 mouse models of insulin resistance — ob/ob, GLUT4^+/–^, and mice with high-fat diet–induced obesity — exhibited increased islet vessel area and decreased intraislet vessel density that were exclusively observed within the β cell core, suggesting islet capillary expansion mediated by dilation (14). Capillary dilation was similarly seen in ob/ob mice, whereas such a change was blunted in db/db diabetic mice with reduced islet blood flow (15). Changes in gene expression of endothelial dysfunction markers were reported in db/db mice (15–17) and high-fat-feeding mice (18) as well as Goto-Kakizaki rats (19) and Zucker diabetic rats (20). Studies of human pancreas specimens reported hypervascularization of islets in T2D subjects as fold changes: 1.36-fold (21), 1.3-fold (18), and 1.18-fold (22).

In the present study, we aimed to examine volumetric intraislet capillary density (vICD) comparatively between non-diabetic (ND) and T2D subjects using large-scale image capture of an entire thick tissue slice in 3D. To this end, we first demonstrated our rationale for quantifying ICD volumetrically in 3D over more commonly used 2D approaches, emphasizing that 3D more effectively captures complex vascular networks which in turn improves statistical analysis. Second, we measured vICD across a gradient of islet sizes in 5 species to learn the fundamentally conserved nature of islet vascularization. Then, vICD and volumetric exocrine capillary density (vECD) were comparatively examined between ND and T2D subjects. Lastly, the current study on vICD led us to define the smallest size of the vascularized islet as being approximately 40 μm in effective diameter. Endocrine structures smaller than this cutoff are non-islet singlets and small clusters of endocrine cells, which we propose to abbreviate as niSSCs. This definition of the islet is momentous, particularly when recent studies on T1D emphasize niSSCs and small islets as the most vulnerable to autoimmune response (23–25). Since there has been no clear definition of the smallest size of the islet, a new term, “endocrine objects,” is being promoted to replace the historical and greatly respected term “islet of Langerhans” (26).

## Results

### Approach to visualizing and analyzing islet microvasculature.

**Figure 1:**
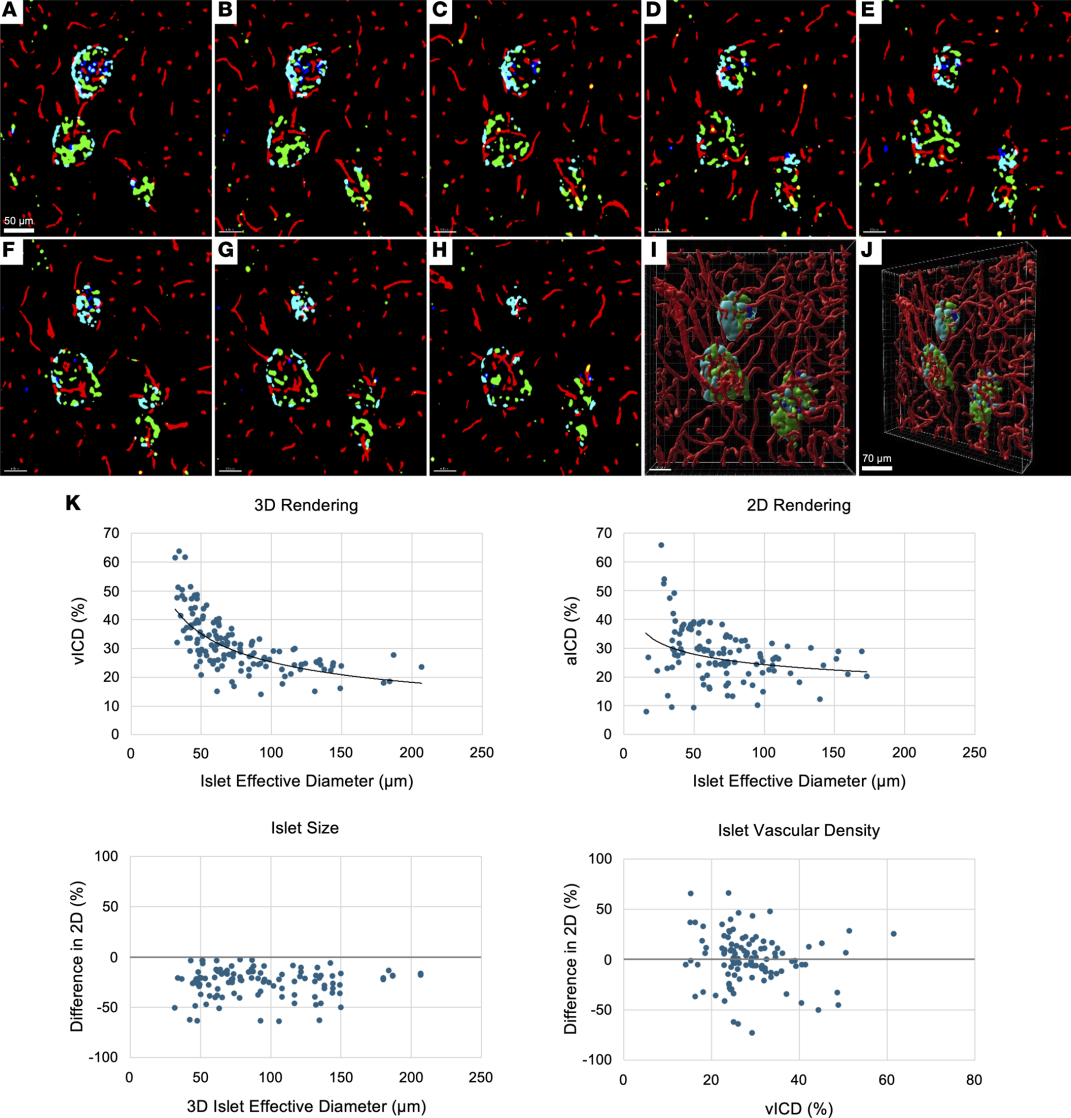
Approaches to visualizing and analyzing islet microvasculature. (**A**–**H**) 2D versus 3D analysis. A series of 2D optical panels of human pancreatic tissue: insulin (green), glucagon (cyan), somatostatin (blue), and CD31 (red). A part of a stack of sequential images (out of a total of 25 optical panels with 5 μm increments) is shown. (**I** and **J**) 3D reconstruction of a stack of 2D images. Scale bars: 50 μm, except the last panel, 70 μm. (**K**) Comparison of data acquired from the same islet sample (~120 islets) using 3D and single-slice images (8 μm) to simulate 2D. Top: 3D image more clearly reveals the relationship between islet size and islet vascularization. Bottom left: Comparison of islet effective diameter between simulated 2D islets and their 3D counterparts, indicating that 2D consistently underestimates islet effective diameter. Bottom right: Comparison of vICD in simulated 2D islets versus 3D indicates inaccuracy in measurement of intraislet vascular density when the full vascular network is omitted.

Historically, the measurement of ICD has been done in 2D. Some studies determined intratumoral microvascular density by counting the endothelial cell lining and quantifying ICD as the number of capillary segments per unit of islet area (12, 14). The importance of 3D volumetric quantification of ICD is demonstrated in Figure 1. Here, we attempted to simulate the common 2D-based analysis using thinly cut tissue sections, utilizing our own resources, with which several optical panels are extracted from a stack of *Z*-axis confocal images (out of a total of 25 panels; Figure 1, A**–**H), on which blood vessels can only be captured as dots and short lines. This issue can be overcome by surface rendering of tubular structures in 3D (Figure 1, I and J), which was especially useful in our measurements of intraislet capillary volume. The challenges in accurately capturing vascular networks with 2D imaging are also reflected statistically. Using the same islet sample (~120 islets) for each approach, we compared islet effective diameter and vICD between islets rendered from a full 3D tissue section (6-μm steps) and a single optical panel simulating 2D sampling. As evidenced below, vICD was highly heterogeneous with respect to islet diameter and decreased predictably as islet size increased (Figure 1K, top). While these relationships were preserved in 2D, they were measurably altered, with individual data points showing a markedly broader distribution than those derived from 3D imaging (Figure 1K, top right). Mean islet diameter and vICD also proportionately decreased by about 9% under the 2D approach for the same islet sample. Comparing individual 2D islets with their 3D counterparts revealed a mean proportional change of –25.2% in islet diameter (Figure 1K, bottom left) and ±18.5% in vICD (Figure 1K, bottom right). The reduced islet diameter measurements in 2D are consistent with the effects of sampling spherical structures at variable depths, where sectioning islets away from the central plane can yield smaller apparent diameters. Similarly, intraislet capillary density derived from single optical planes showed notable variability in comparison with individual 3D islet reconstructions, as indicated in Figure 1K, bottom right. However, simulated 2D intraislet capillary density was not consistently greater or less than 3D vICD between reconstructions of the same islet, hence the reporting of its mean proportional change as ±18.5%. This absence of a consistent directional bias in the variability of 2D intraislet capillary density aligns with the appearance of 2D vessels in Figure 1, A–J, which suggests that single cross sections of intraislet capillary networks capture partial representations of the full vasculature network. Therefore, volumetric 3D analysis may better represent islet morphology and vasculature architecture.

### 3D reconstruction of islets and their microvasculature.

**Figure 2:**
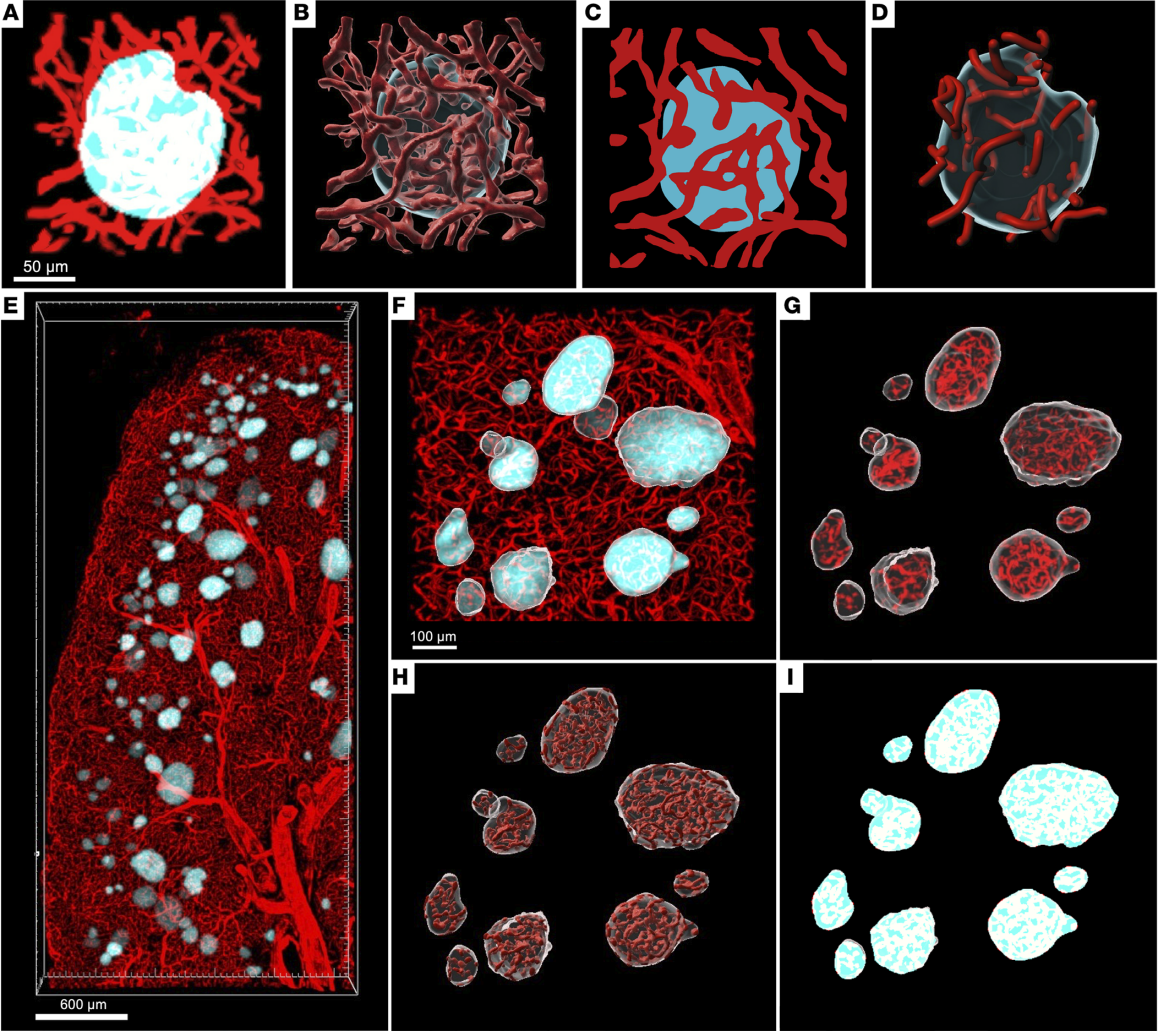
3D reconstruction of islets and their microvasculature. (**A**) Top-down 3D fluorescent view of a region of 50 μm depth. Islet cell marker (HPi1; cyan), CD31 (red). Scale bar: 50 μm. (**B**) Islet and vasculature generated as 3D surfaces. (**C**) Compressed top-down 3D view of rendered capillaries. (**D**) Rendered capillaries isolated at the islet periphery. (**E**) Workflow of vICD measurement. Large-scale view of a specimen. Scale bar: 600 μm. (**F**) Fluorescent image. (**G**) Islets with intraislet capillary fluorescence isolated. (**H**) Intraislet vasculature generated as 3D surfaces. (**I**) Binary fluorescence masked to rendered structures and later used for vICD determination. Scale bar: 100 μm.

The integrated nature of the endocrine (i.e., islets) and exocrine pancreas is revisited in Figure 2, A–D. 3D views of immunofluorescence staining (Figure 2A) and a rendered islet with nearby capillaries (Figure 2B) demonstrate the integration of islet microvascular networks. This integration can be viewed more closely in a top-down view of rendered islets and their capillaries (Figure 2C), as well as with filtering of capillaries that appear directly along the islet margins (Figure 2D). The workflow of vICD measurement is shown in Figure 2, E**–**I. The degree of islet vascularization was quantified by vICD relative to total islet volume, using a large-scale image capture method (Figure 2E). Islet structures were first rendered using fluorescence from islet cell staining (Figure 2F; HPi1 in cyan). Endothelial cell signals (Figure 2G; CD31 in red) within the islets were then isolated, allowing specific rendering of intraislet capillary networks (Figure 2H). Binary fluorescent channels were masked to these structures (Figure 2I), which enables quantification of vICD as the percentage of 3D pixels, or voxels, within an islet that are occupied by capillaries. Application of this analysis to a large number of islet samples reveals previously unreported vascularization patterns across the gradient of islet sizes. Thus, 3D imaging offers not only a clearer physical representation of islet vascularization, but also more efficient and robust quantification. This makes it a powerful tool with which to unveil vascular morphology in the pancreas.

### Characteristics of vICD among various species.

We first aimed at defining the fundamental characteristics of vICD. Previous reporting of ICD has followed assumptions that islet vascularization should be homogeneous regardless of islet size (14, 21, 27), leaving the potential relationship between islet size and vascularization minimally understood. A 2017 study reported mean volumetric microvascular densities of about 9% in human islets and about 21% in mouse islets (28). Although this was intended to be a 3D analysis, these findings could have been due to thin slice preparation (120 μm) and assessments of islets greater than 225 μm in diameter. Inclusion of small islets (e.g., 40–50 μm) is important in studying various parameters of islets.

**Figure 3:**
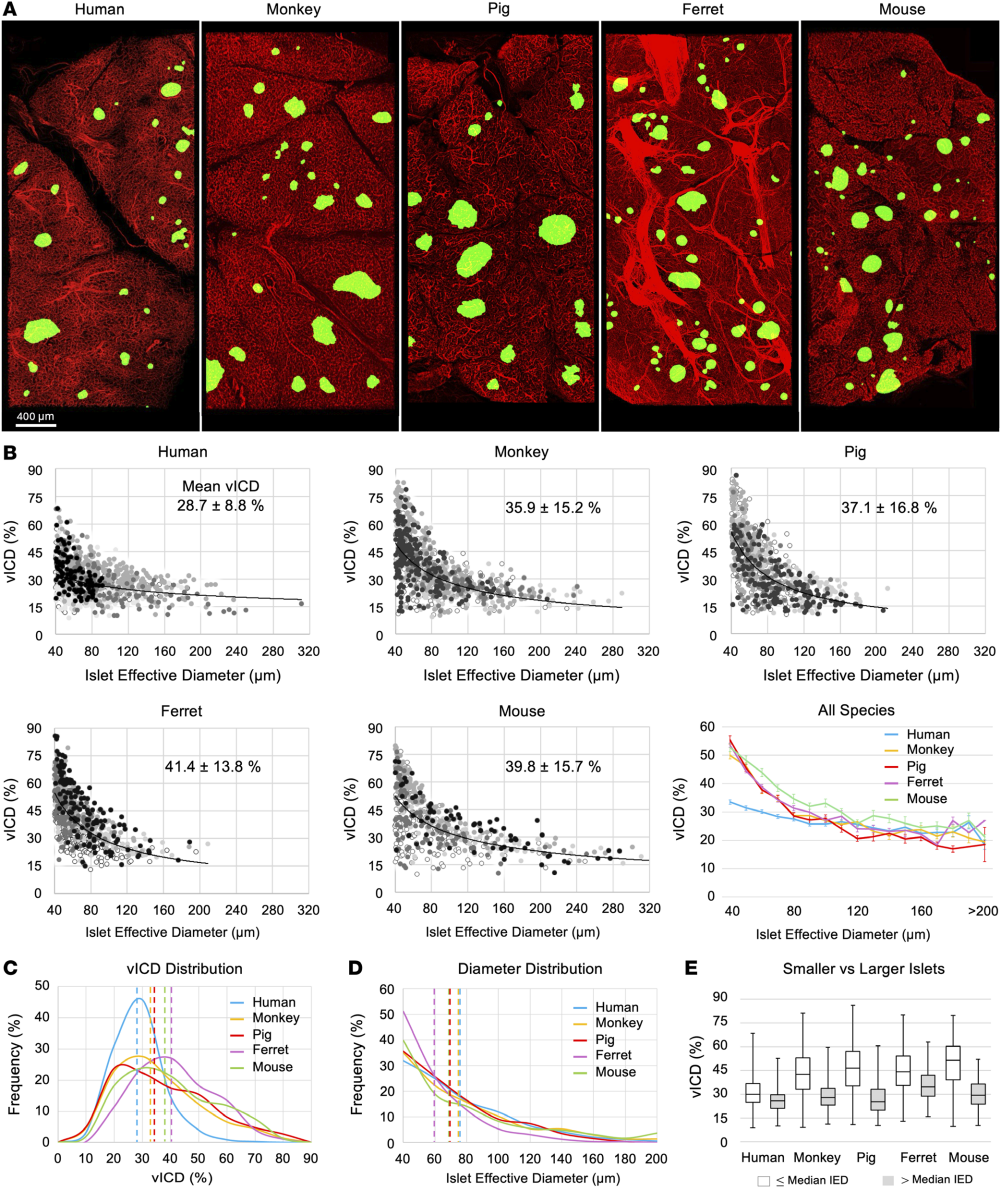
Characterization of vICD in various species. (**A**) Representative fluorescent 3D images from each species analyzed. Islet cell marker (HPi1) or insulin (green), CD31 or tomato lectin (red). Scale bar: 400 μm. (**B**) Percentage vICD plotted against islet effective diameter (μm). Combined data from 5 individuals sampled (indicated as 5 shades of circles from black to white). Bottom right: Mean vICD across islet diameter in all species. All animal species exhibited significantly greater mean vICD compared with humans (*P* < 0.001, effect size 0.60–1.14). (**C**) Interspecies comparison of vICD density distribution. Median vICD for each species is indicated by the dashed lines. (**D**) Interspecies comparison of islet effective diameter distribution with median diameter values also indicated by dashed lines. Legends are included in the plots. (**E**) The difference in vICD between islets with islet effective diameter (IED) smaller and greater than median islet effective diameter of each species. Median vICD is shown. Mean vICD was significantly different between smaller and larger islets in all 5 species (*P* < 0.001, effect size 0.46–1.41).

In the present study, vICD was quantified in 5 species with 5 subjects per species: human (total islet number: *n* = 1,169), monkey (*n* = 847), pig (*n* = 770), ferret (*n* = 859), and mouse (*n* = 557). Their biological background information is given in Table 1 (human) and Table 2 (all other species). Images of immunofluorescently stained thick pancreatic tissue slices from these species are shown in Figure 3A (islets in green and vasculature in red). Plotting vICD against islet effective diameter revealed conserved heterogeneous distribution of vICD across islets of varying size (Figure 3B). Note that each differently shaded collection of data points represents an individual subject within the dataset. Both mean vICD and the observed variation in vICD for islets at a given size gradually decreased as islet size increased. Capillary volume increased linearly with islet volume, but the ratio of capillary to islet volume decreased, which led to this relative reduction of vascular density in larger islets. The variability in vICD was markedly higher in small islets compared with large ones; however, mean values converged to a common density of approximately 20% among all species, as shown in the plot in Figure 3B comparing trends in vICD between the species. Mean vICD trends across islet diameter in all species are also shown.

All animal species exhibited significantly greater mean vICD compared with humans (*P* < 0.001), further supported by strong effect size (0.60–1.14). Comparison of mean vICD in human (28.7% ± 8.8%) and mouse (39.8% ± 15.7%) pancreata indicates that mouse vICD was higher by a factor of 1.33, as opposed to the 2- to 5-fold difference previously reported (12). Mean vICD values in monkeys (35.9% ± 5.2%), pigs (37.1% ± 16.8%), and ferrets (41.4% ± 13.8%) were also significantly greater than human vICD by factors of 1.25, 1.29, and 1.44, respectively. The present findings are consistent with previous findings that mice have higher intraislet capillary density than humans (21, 28, 29). Human and monkey vICD appeared normally distributed, whereas pig, ferret, and mouse vICD exhibited slight bimodal distribution as shown in Figure 3C. In light of the relationship between vICD and islet size, interspecies differences in vICD may be partially explained by differences in the distribution of islet size (Figure 3D). For instance, ferrets had the lowest mean islet diameter of the 5 species examined, and since small islets had higher vICD on average, this may contribute to their mean vICD being the highest among all species. However, this trend was absent among mice, as they exhibited the greatest mean islet diameter but the second greatest mean vICD. All 5 species showed significant differences in vICD (*P* < 0.001) between islets smaller and larger than median diameter, as supported by a moderate effect size for humans (0.46) and large effect sizes for animal species (1.20–1.41) (Figure 3E). Over a decade ago, we carried out a comparative study among several species (human, monkey, pig, rabbit, and mouse) and showed that their islet size distributions fall into a similar range, despite marked interspecies differences in overall body and pancreas size, as well as total β cell mass (30). Previous data suggested a size limit (approximately several hundred micrometers in diameter) past which islets are no longer functional. This fundamental characteristic was also observed in the current study. Islets larger than the median diameter value contributed roughly 90% (precisely in the range of 86.9%–95.7%) of all islet volume among the species but, following aforementioned trends, were far less densely vascularized.

### Comparison of vICD between ND subjects and patients with T2D.

**Figure 4:**
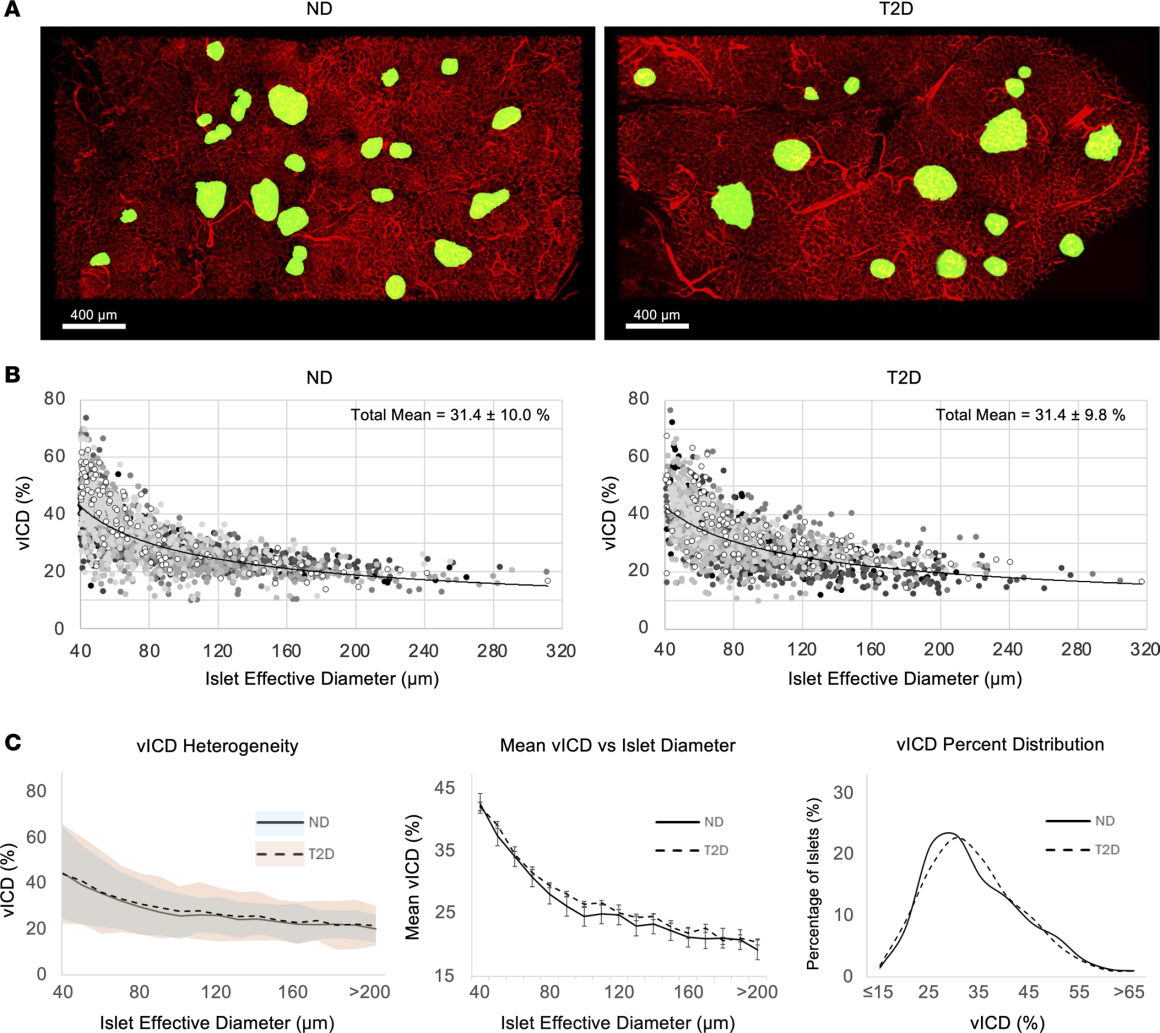
Comparison of vICD between ND and T2D human subjects. (**A**) Representative fluorescent 3D images from ND and T2D human subjects. Islet cell marker (HPi1; green), CD31 (red). Scale bars: 400 μm. (**B**) Distribution of vICD (percent) along with islet effective diameter (μm). ND and T2D human mean vICDs were not significantly different (*P* = 0.66). (**C**) Comparison of vICD features between ND and T2D subjects.

Based on the foundation of islet vascularization established through the species comparison, we examined pancreatic tissues from ND and T2D subjects consisting of 8 age- and sex-matched pairs: 4 male and 4 female pairs; age range of 35–65 years; various durations of T2D from 0 to over 15 years (Tables 3 and 4). Strikingly, regardless of these individual differences, no significant difference in mean vICD was found between ND (31.3% ± 9.9%) and T2D subjects (31.1% ± 9.5%) (*P* = 0.66) (Figure 4A). These findings stand in direct contrast to previous 2D-based analyses reporting significantly increased ICD in T2D (18, 21, 22). All pancreatic tissue samples showed profound heterogeneity in islet size, vICD, and spatial distribution as shown in Figure 4B. As noted in the species comparisons, vascularization in ND and T2D islets decreased and became less variable with increasing islet size. Further comparison of vICD distribution, frequency, and variation between two groups exhibited closely comparable features (Figure 4C). Notably, there were differences in organ donors’ BMI between the two groups (Tables 3 and 4). In individuals with high BMI, it may be expected that intrapancreatic fat could confound the results. However, it appeared that adiposity did not affect vICD in those particular subjects.

### Comparison of peri-islet vECD and interface capillary counts between ND and T2D subjects.

In the obese and diabetic db/db mouse model, peri-islet acinar cells were found to be significantly larger than tele-islet ones and exhibited a zonated gene expression signature of upregulated trypsin genes and mTOR activity (31). This was proposed to be induced by islet-derived cholecystokinin, suggesting the involvement of exocrine-endocrine crosstalk. This led us to question, under the integrated, thus continuous, pancreatic blood flow, how vascularization at the interface of the endocrine and exocrine pancreas may change with islet size and T2D pathogenesis.

**Figure 5:**
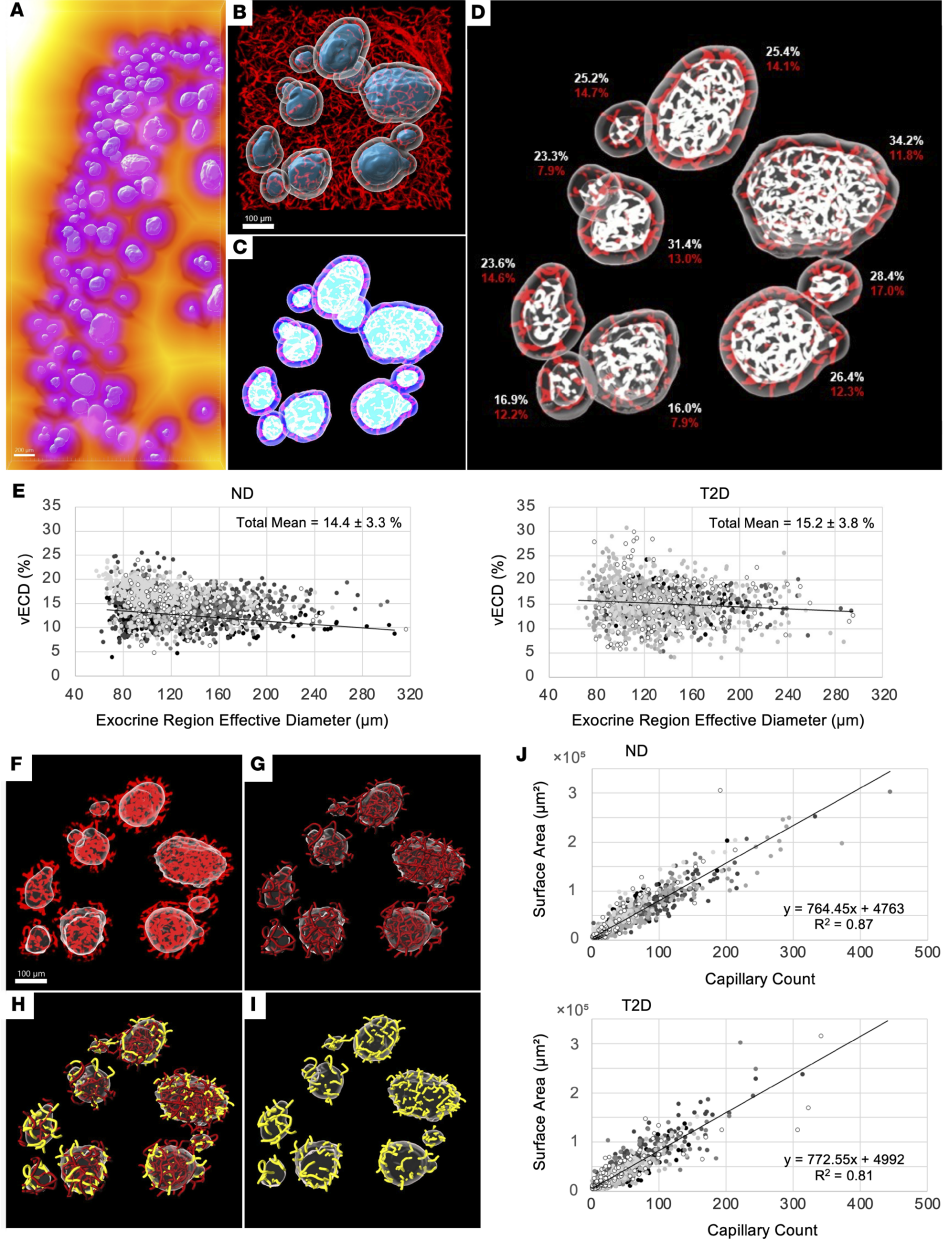
Comparison of peri-islet vECD and interface capillary counts between ND and T2D human subjects. (**A**) Approach to volumetric exocrine capillary density (vECD). Identification of exocrine regions in the immediate periphery of islets based on distance between voxels. Purple indicates close proximity to islets. Scale bar: 200 μm. (**B**) Exocrine regions (transparent) 25 μm thick rendered in 3D immediately outside the islets (opaque blue). Capillaries are in red. This thickness was set so that exocrine region diameter would occupy a diameter range similar to that of islets. (**C**) Binary fluorescence for exocrine capillaries (red), exocrine regions (purple), and islets (cyan). (**D**) vICD values and intraislet capillaries in white, vECD values and exocrine capillaries in red. (**E**) Plots of vECD against islet effective diameter in human subjects with and without T2D. Mean peri-islet vECD values were significantly different (*P* < 0.001), but effect size (0.23) indicated minimal practical significance. (**F**) Approach to quantifying the number of individual capillaries at the islet periphery. Capillary fluorescence inside and 25 μm outside of rendered islets. Scale bar: 100 μm. (**G**) Rendered capillary segments. (**H**) Highlighted segments at the periphery of islets, intersecting their margins. (**I**) Isolation of highlighted segments for quantification. (**J**) Plots of islet surface area against the number of capillaries at the islet periphery in human subjects with and without T2D. Mean islet surface area to segment ratios (μm^2^/segment) were significantly different (*P* < 0.01), but effect size (0.11) indicated minimal practical significance.

Exocrine regions were rendered directly outside the islets using embedded information regarding how far each 3D pixel, or voxel, was from a central object or objects (Figure 5A). As shown in 3D (Figure 5B), exocrine regions were generated where their bounds began immediately outside the islet margins (blue) and terminated 25 μm outside the islets (translucent white), as this cutoff value provided a range of effective exocrine region diameters (~60 to ~300 μm) comparable to the range of effective islet diameters observed (~40 to ~300 μm). Narrower exocrine region widths were attempted but resulted in more sporadic peri-islet vICD, prompting use of 25 μm as an outer bound. In a process analogous to vICD determination, capillaries were isolated for rendering of exocrine capillary networks. Binary fluorescent channels were masked to the exocrine regions and their capillaries (Figure 5C), enabling quantification of peri-islet vECD. The vICD and vECD of islets and their corresponding exocrine regions are displayed in Figure 5D. As shown in Figure 5E, peri-islet vECD was plotted against exocrine region diameter to facilitate comparison with vICD.

Although a significant difference was found in mean peri-islet vECD (*P* < 0.001) between ND (14.4% ± 3.3%) and T2D (15.2% ± 3.8%) exocrine tissues, an effect size of 0.23 indicates that the practical difference between the mean values is relatively inconsequential when adjusting for large islet sample size. An absolute difference of less than 1% between these mean values, as well as the plots of peri-islet vECD against exocrine region effective diameter (Figure 5E), reinforces the notion that this difference in vascular density would be unlikely to notably impact overall exocrine vascular integrity. However, given the context that these quantifications came from the immediate islet periphery, this small but present difference in vECD between ND and T2D suggests that more profound differences in vECD may appear further from the islets. Evaluation of more distant vECD was not completed for the present study because of difficulty in identifying a systematic method of rendering distant exocrine regions, but will likely be of interest in future investigations.

Unlike vICD with islet diameter, vECD did not appear to have a discernible relationship with exocrine region diameter. This indicates that the size-dependence of vICD is likely a specialized feature of islets. Present vECD findings confirm previous conclusions that endocrine tissues are twice as densely vascularized as nearby exocrine tissues (32, 33), as opposed to an overestimated difference reported by 2D analyses (2, 4, 34). A previous study using whole-mount immunohistochemistry and 3D image analysis showed visibly lower capillary density in the exocrine tissues but did not confirm the extent of this difference through quantification (35).

To further confirm the integrated pancreatic blood flow between the endocrine (islets) and exocrine pancreas that we had proposed (5–8), we used a new image analysis tool, machine learning capillary segmentation, and directly counted vascular entry/exit points in each islet. Counting capillary segments that intersected the endocrine-exocrine interface revealed a strong linear relationship between islet surface area and vascular integration. As islet surface area increased, the number of capillary segments that crossed the islet margins did as well. Isolation and quantification of intersecting segments are shown in Figure 5, F**–**I, and the relationship between these segment counts and islet surface area is shown in Figure 5J. Despite having the lowest mean capillary density, the largest islets of each individual, ND or T2D, consistently demonstrated the highest count of capillary segments along their margins. This is the same pattern demonstrated in the relationship between total intraislet capillary volume and islet volume.

The mean islet surface area per segment ratio (μm^2^/segment) was significantly different between ND (1.12 × 10^3^) and T2D (1.23 × 10^3^) human subjects (*P* < 0.01) but not practically different per a small effect size of 0.11. Further, the slope of islet surface area against capillary count was highly comparable between ND (764.5 μm^2^/segment) and T2D (772.6 μm^2^/segment). The strength of the relationship between islet size and capillary integration therefore did not appear to be altered in T2D. A lack of differences in vECD and endocrine-exocrine microvascular integration suggests that capillaries in ND and T2D pancreata are highly comparable outside the islets, not solely inside. Though vascular density directly evaluates the capacity of an islet to be perfused, it will be beneficial for future investigations to assess whether islet blood flow itself is altered in T2D in vivo. Atherosclerotic changes or altered vessel permeability, unexamined by the present study, may complicate blood flow despite normal pancreatic vascular densities and distributions in T2D.

### Lobular distribution of islets.

**Figure 6:**
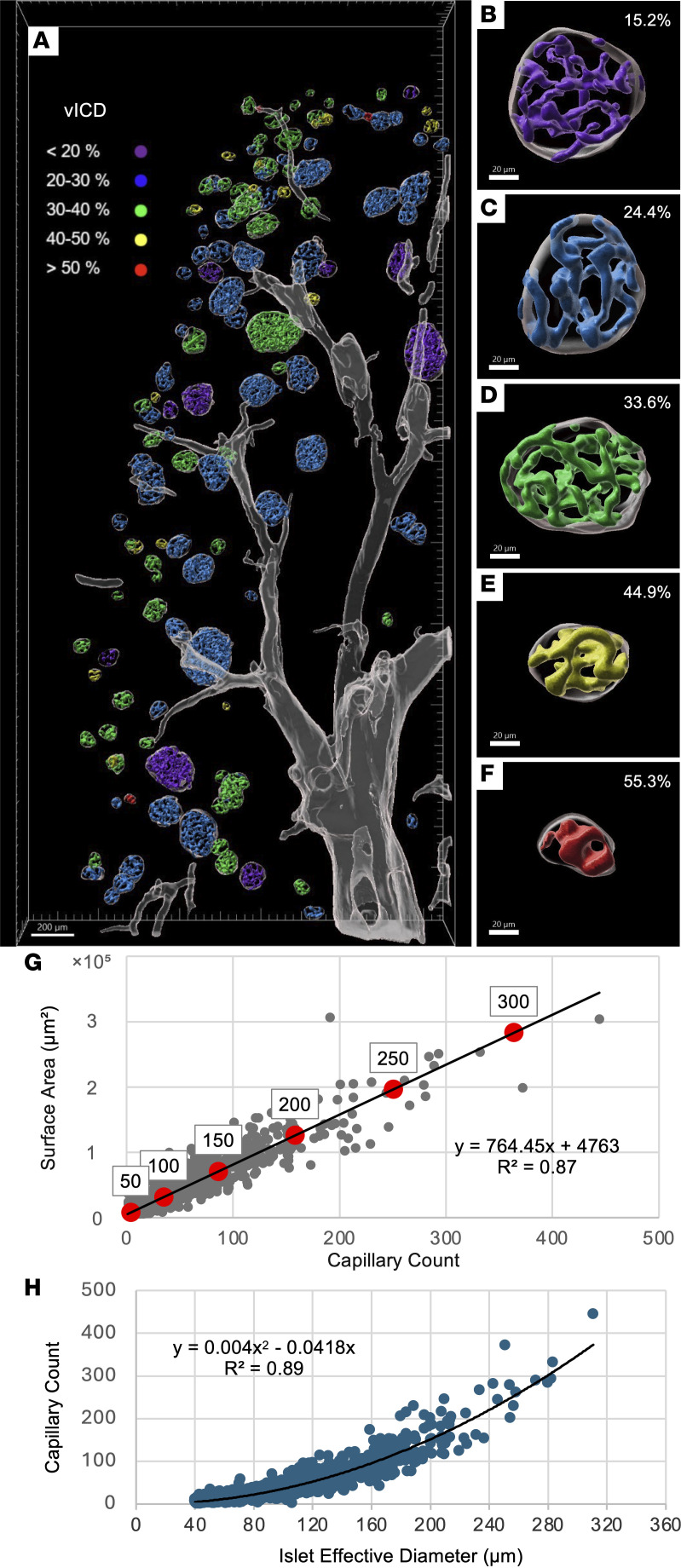
Lobular distribution of islets. (**A**) Rendered islets and capillaries within pancreatic tissue slice of a 24-year-old ND man (BMI 29.3). Smooth muscle actin–containing large blood vessels including arterioles are shown in transparent white. Islet capillary networks are color-coded based on vICD. (**B**–**F**) Representative islets with different vICD. (**G**) Count of segments on the islet margins plotted against islet surface area. Numerically labeled red data points indicate islet effective diameters (μm). (**H**) Conversion of the plot in **G** to demonstrate the relationship between islet margin segment count and islet effective diameter.

A large-scale view of islet distribution with regard to vICD is shown in Figure 6A. Islets are stratified and color-coded by their vascular densities as <20% in purple, 20%–30% in blue, 30%–40% in green, 40%–50% in yellow, and >50% in red. Representative islets of each density category are featured in Figure 6, B–F. As was consistently demonstrated in our analysis, islets with denser vasculature are typically smaller in size. This lobular spatial disposition of islets confirms our previous report that larger islets were more closely associated with arterioles and smaller islets were prominently found in the periphery (7). Other than highly vascularized small islets, there appears to be no explicit pattern of distribution attributable to vICD.

The analysis of capillary counts along the islet border in Figure 5J provides the average number of capillaries in relation to islet diameter (Figure 6G). We found a linear relationship between the number of capillaries and islet surface area, which was expected under the integrated pancreatic blood flow. To offer a general sense of the measurement, among 1,800 islets in Figure 6G (diameter 40 to 310 μm), the average islet (~90 μm) had 35 capillaries on its interface, whereas an islet 200 μm in diameter can be expected to have approximately 155 segments. Note that blood flow directions (i.e., islet to exocrine pancreas and vice versa) were 1:1 as we have previously reported based on intravital recording of islet red blood cell flow in mice (5).

Furthermore, in view of recent emerging interest in singlets and small clusters of pancreatic endocrine cells (23, 24), we attempted to define a clear distinction between these cells and the islets. Since it has long been described that the islet is highly vascularized, we reasoned that one important criterion should be a presence of capillaries. The measurement of islet interface segment counts in over 3,000 islets revealed that groups of endocrine cells experienced direct vascularization at a diameter of approximately 40 μm. Conversion of the *y*-intercept value in Figure 6G from surface area to diameter, assuming a generally spherical shape for islets, supports this diameter value. As shown in Figure 6H, islets gradually decreased in vascular integration as they decreased in size, with groups of endocrine cells ceasing to be directly vascularized around the aforementioned diameter. In our qualitative analysis, we noted regional vascularization of smaller endocrine cell clusters with single capillaries having occasional contact with cell cluster margins; however, direct vascularization was absent.

## Discussion

The present study highlights the advantages of 3D spatial analysis of the network of tubular structures such as vasculature. As we simulated, extrapolation of the data of one optical slice to the entire islet microvasculature is insufficient. These filamentous structures appear in one slice but not another, and capillaries passing through a slice appear only as dots and short lines. The analysis of five species revealed the complexity of islet microcirculation, reflected as intra-individual, intra-species, and inter-species heterogeneity. Capillary volume gradually increases collinearly with islet volume, which results in larger islets having reduced vICD compared with smaller islets. Large islets, which were defined as having diameter greater than median islet diameter, accounted for approximately 90% of total islet volume with minor variation between species. The markedly higher degree of vICD heterogeneity in smaller islets, as observed in all plots of vICD against islet diameter, may be explained by the disproportionate effect of differences in capillary volume on islets of smaller volume. Notably, all species exhibited profoundly similar heterogeneity, suggesting that islet vascularization is highly conserved.

The lack of appreciable differences in vICD, vECD, or interface capillary integration between ND and T2D subjects raises questions regarding the notion of islet vascular involvement in the pathogenesis of T2D (36–38). Magnetic resonance imaging studies of patients with T2D reported decreased pancreas size, increased pancreatic fat content, and an irregular morphology in comparison with ND subjects (39–41). Further, such serrated pancreas margins have been associated with advanced peripheral microvascular complications (40). Owing to the gradual loss of β cell function in T2D, it has been proposed that pancreatic microvasculature is not only affected but also implicated in T2D endocrine dysfunction (18, 21, 22). Various 2D morphometric analyses have suggested that islet microvasculature is in fact changed in T2D (18, 20, 21, 41–43), and intra-pancreas fat deposition would suggest intra-pancreas insulin resistance with local vascular consequences (44).

In light of new understandings of islet vascularization, interface segmental capillary analysis has also revealed criteria for what may constitute an islet with vascularization, which distinguishes it from singlets and small clusters of islet cells. Below a diameter of approximately 40 μm, groups of endocrine cells demonstrate far less direct vascularization, relying at most on blood flow from capillaries they have contact with but are not directly intersected by. As demonstrated by direct measurement of the number of interface capillary segments, capillary integration is consistent in endocrine cell clusters greater than 40 μm in diameter.

Integrated blood flow was first proposed based on two experimental methods: (a) in vivo mouse intravital recording of fluorescently labeled red blood cell circulation; and (b) structural analysis of pancreatic vasculature in 3D (5, 7). It exemplifies the case that functional studies provide insight into dynamic activities, while structural analysis offers context necessary to interpret physiological processes accurately. Integrating both approaches allows for a comprehensive understanding of systems, often revealing correlations that may not be readily apparent. The current study further confirms that the anatomical and mechanistic viewpoints in conjunction can enrich the overall understanding of complex biological systems.

## Methods

### Sex as a biological variable.

Our study examined male and female humans, and no sex-dimorphic effects are reported. Both sexes of monkeys, ferrets, and mice were examined; however, because of the small sample size in either sex, the results are not conclusive regarding sex dimorphism. Pigs were exclusively female owing to availability; therefore, it is unknown whether the findings are relevant to male pigs.

### Human pancreas specimens.

Human pancreata from donors were provided by the Gift of Hope Organ Procurement Organization (Chicago, Illinois, USA). Written informed consent from a donor or the next of kin was obtained for use of samples in research.

### Animal pancreas specimens.

Monkey, pig, and ferret pancreata were provided by the Carlson Veterinary Clinic of the Animal Resource Center.

### Antibodies.

The following primary antibodies were used: mouse monoclonal anti–pan-endocrine (AB_1625452, HPi1, Novus Biologicals), mouse monoclonal anti-insulin (Research Resource Identifier [RRID] AB_2811080, Novus Biologicals), and mouse monoclonal anti–human CD31 (AB_314328, BioLegend). The primary antibodies were conjugated with a combination of amine-reactive fluorophores (*N*-hydroxysuccinimide esters, Thermo Fisher Scientific). DyLight 594–labeled tomato lectin from *Lycopersicon esculentum* (AB_2336416, Vector Laboratories) and mouse monoclonal anti–actin α-smooth muscle–Cy3 conjugate (AB_476856, Sigma-Aldrich) were used.

### 3D pancreas imaging.

The detailed method for 3D pancreas imaging was previously described (45, 46). Briefly, a frozen pancreas tissue block (~5 mm in thickness) was fixed in 4% paraformaldehyde, embedded in 2% agarose gel, and mounted on a vibratome. Sections (600–800 μm in thickness) were collected in cold PBS. These macrosections were then immunohistochemically stained overnight. Optical clearing was carried out by sequential incubation with 20%, 50%, 80%, and 100% (wt/vol) solutions of d-fructose and 0.3% (vol/vol) α-thioglycerol (Sigma-Aldrich) for 2 hours each and overnight in the last solution at 34°C with gentle agitation. Leica SP8 and Stellaris 8 laser scanning confocal microscopes (Leica Microsystems) were used to image tissue slices mounted between coverslips using a ×10 objective.

### Processing and reconstruction.

After conversion to TIFF format in Fiji, image processing and 3D reconstruction were carried out using Imaris 10.2 (Bitplane) software. Pixels were 1.82 by 1.82 μm, and step size was 5–6 μm. A Gaussian blur of 3–5 μm and a background subtraction filter of 300 μm were applied to islet-relevant fluorescent signals. Regarding vasculature-relevant fluorescence, a Gaussian blur of 1.25–2 μm was applied, and background subtraction was circumstantial but fell in the 12–15 μm range. Gaussian blurs were applied first during image processing to avoid false detection and removal of foreground. 3D reconstruction of islets and capillaries followed the pipelines outlined in Figures 2 and 5. All masked channels that were generated for the isolation of select structures were not modified beyond the original image processing, though this, by necessity, does not include binary channels generated for volumetric analysis at the end of the image analysis pipeline. Accidentally conjoined islets were manually separated during analysis, and islets omitted by the surface creation wizard were individually reconstructed with either a native marching cube algorithm or a manual tracing tool using local fluorescence.

### Statistics.

Data were imported to a Microsoft Excel template for evaluation of vICD, vECD, and islet marginal segment counts. Parameters were analyzed for density distribution, normality, and distribution with respect to islet effective diameter. Statistical outliers were isolated directly within Imaris 10.2 to confirm their status as true or artifact. After 2-tailed *t* tests and 2-way ANOVA at α values of 0.01 and 0.001, Cohen’s *d_s_* was used to adjust for large islet sample size (hundreds to thousands) and evaluate the practical significance of intergroup differences determined to be statistically significant.

### Study approval.

All the procedures involving animals were approved by the University of Chicago Institutional Animal Care and Use Committee. The use of deidentified human tissues in the study was approved by the Institutional Review Board at the University of Chicago.

### Data availability.

The datasets generated and/or analyzed during this study are available upon reasonable request. Values for all data points in graphs are reported in the Supporting Data Values (supplemental material available online with this article; https://doi.org/10.1172/jci.insight.202118DS1).

## Author contributions

MH conceived the idea and designed the study. AMT, FRM, and MH performed experiments and analyzed data. AMT and MH wrote and revised the manuscript. All authors contributed manuscript editing. MH is the guarantor of this work and, as such, had full access to all the data in the study and takes responsibility for the integrity of the data and the accuracy of the data analysis.

## Conflict of interest

The authors have declared that no conflict of interest exists.

## Funding support

This work is the result of NIH funding, in whole or in part, and is subject to the NIH Public Access Policy. Through acceptance of this federal funding, the NIH has been given a right to make the work publicly available in PubMed Central.

- National Institutes of Health (NIH) DK138473, DK117192, and DK127786, as well as DK020595 to the University of Chicago Diabetes Research and Training Center (Physiology Core) to MH.
- A gift from the Kovler Family Foundation to MH.

## Supplementary Material

Supporting data values

## Figures and Tables

**Table 3:**
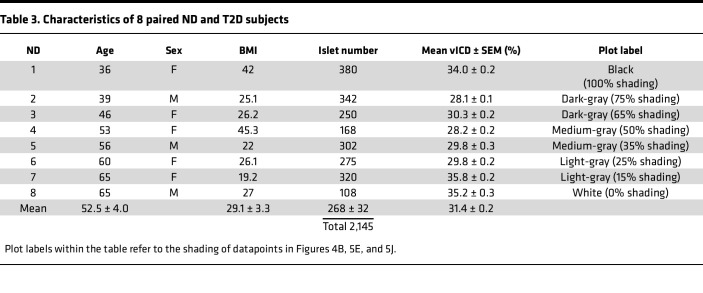
Characteristics of 8 paired ND and T2D subjects

**Table 1:**
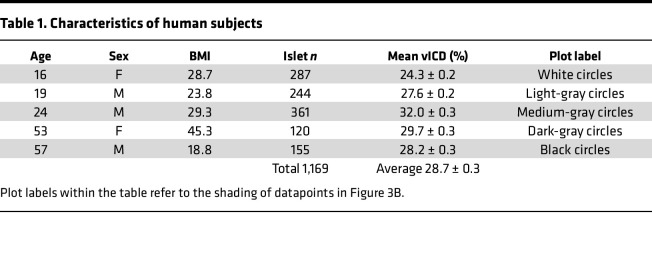
Characteristics of human subjects

**Table 2:**
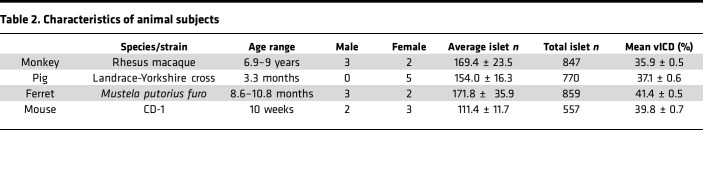
Characteristics of animal subjects

**Table 4:**
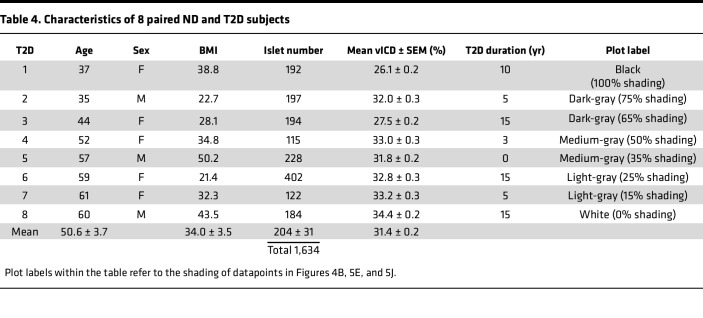
Characteristics of 8 paired ND and T2D subjects

## Version 1 04/23/2026

In-Press Preview

## Version 2 06/22/2026

Electronic publication
